# A Comparison of the Effectiveness of Surgical and Nonsurgical Treatment of Legg-Calve-Perthes Disease: A Review of the Literature

**DOI:** 10.1155/2012/490806

**Published:** 2012-08-16

**Authors:** Mohammad Taghi Karimi, Tony McGarry

**Affiliations:** ^1^Musculoskeletal Research Centre, Isfahan University of Medical Sciences, Isfahan, Iran; ^2^National Centre for Prosthetics and Orthotics, University of Strathclyde, Glasgow G1 1XQ, UK

## Abstract

Legg-Calve-Perthes disease (LCPD) is a degenerative condition of the hip joint characterised by idiopathic avascular necrosis of the femoral head. Loss of bone mass causes a degree of collapse of the joint and may result in deformity of the ball of the femur and the surface of the hip socket. A reduction in hip joint range of motion, alternation in growth of femoral head, and associated pain are most important problems associated with this disease. Various treatment methods are currently in use and aim to increase containment of the femoral head within the acetabulum, redistribute loading patterns applied to the femoral head, and to decrease the final deformities associated with this condition. These treatments depend on a variety of underlying factors and the aim of this paper was to determine appropriate pathways for treatment and the evidence of treatment method success. A review of the relevant literature was carried out in a variety of data bases including PubMed and ISI Web of Knowledge, and Gale between 1950 and 2011. Research results were categorised depending on the identified treatment method. The success of each treatment pathway was assessed and reasons for the pathway selected were grouped by the age of disease onset, follow-up period, and the final outcome. Evidence relating to the effectiveness of the treatment method used was conflicting. Different methods of screening and follow-up periods were employed in each study which used subjects of varying ages. Minimal evidence of sufficient quality exists in the literature to determine the most appropriate treatment of Legg-Calve-Perthes disease. Research provides some evidence to suggest that nontreatment may be as effective as orthotic or surgical intervention. More research is required to determine the effectiveness of orthotic and surgical treatment.

## 1. Introduction

Legg-Calve-Perthes disease (LCPD) is a condition in which the blood supply of femoral head is interrupted and the bone temporarily dies. This may lead to irritability of the hip joint and, depending on the severity, deformation of the head of the femur. The aetiology of LCPD is idiopathic but is likely to be multifactorial and may be of genetic or deprivation influence [1, 2]. The disease occurs in children between 5 and 12 years of age and is more prevalent in males. Incidence differs in different countries and is also dependant on race (10.8 per 100,000 Caucasian, 0.45 Negroid children) [3, 4]. Higher incidence is recorded in the areas of lower population compared to more densely populated areas [3, 5, 6].

Problems associated with LCPD include decreased hip joint range of motion, especially abduction, and alternation in the growth of femoral bone which may lead to pain while walking. Long term outcomes include instability or reduced range of motion of the hip joint and increased incidence of osteoarthritis [3, 5, 6].

**Figure 1:**
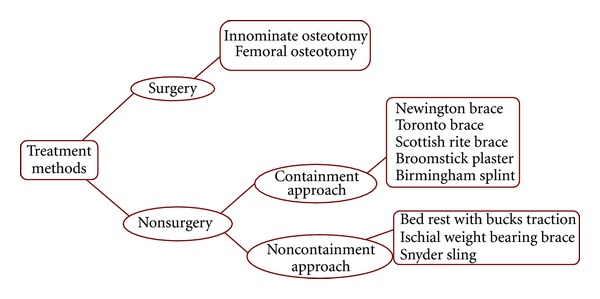
Operative and nonoperative treatment of LCPD.

A variety of treatment methods are used in LCPD to maintain hip joint range of motion and aim to relieve clinical symptoms (especially pain) and contain the hip joint. Treatments may be classified as operative and nonoperative methods (Figure 1).

Although the original description of LCPD was made over one hundred years ago [7], there is still a lack of agreement as to the most appropriate form of treatment of the condition and the patient groups to whom it should be applied. There is no general agreement regarding whether operative or nonoperative treatment is beneficial [10]. As the disease is relatively uncommon, most reports are based on a limited number of patients. The availability of different treatment modalities in various countries also adds uncertainty and increases difficulty in facilitating comparison of the outcome of different treatment methods [11, 12].

Most surgical corrections for LCPD are carried out to increase the containment of femoral head within the acetabulum by femoral or innominate osteotomy [13]. A combination of femoral and innominate osteotomy is also suggested by some researchers [11, 13].

Nonoperative pathways are divided into either containment or noncontainment of the femoral head within the acetabulum.

The containment method was first described by Craig (1957) and later revised by Bobechko et al. (1968) [14]. This method assumes that the most important factor in the treatment of LCPD is to prevent deformity of the femoral head. By containing the femoral head within the acetabulum, the femoral head is protected from compression by the acetabular margin. Containment position is defined as abduction and internal rotation of the extremity until the femoral epiphysis is well inside Perkins line [11, 12].

Additionally, orthotic devices may be used in nonoperative treatment of the condition and are used in both containment and noncontainment treatment methods [7, 11–14]. Different designs are available and include the Newington brace; the Toronto orthosis; the Scottish rite orthosis; the broomstick plaster, the Birmingham orthosis.

Previous review papers exist relating to the nonsurgical treatment of LCPD. Previous reviews are limited by the parameters examined such as type and number of studies. No attempt to grade the quality and study design of literature according to a recognised scale has been undertaken. Furthermore, no assessment of treatment success has been examined and no comparison of the effectiveness the intervention recorded. The aim of this paper is to determine the most effective treatment method based on the outcome achieved by examining results of each category of treatment.

## 2. Method

**Figure 2:**
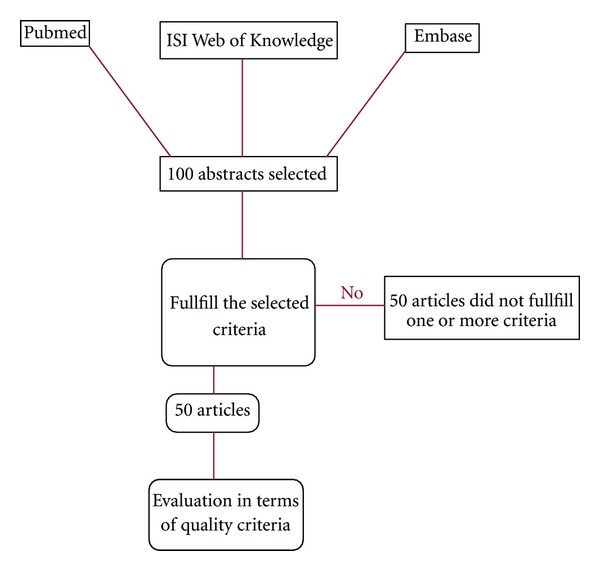
Results of review process.

An electronic search was completed via the Pubmed, Embase, and ISI Web of Knowledge data bases from 1950 to 2010. The key words used for the search were Perthes disease treatment, avascular necrosis of the hip, and included specific topics, such as gait analysis, orthosis, and containment and noncontainment approach, which were identified by a multidisciplinary team of expert scientists and clinicians. The abstract and title of each individual study was assessed by reviewers. A first selection of the relevant articles was completed based on whether or not the title/abstract addressed the key words. Selection stages are illustrated in Figure 2. The second selection of the articles was completed according to the following criteria:

1. articles addressing the Perthes disease and its treatment methods,
2. experimental studies published in English.

Parameters such as the number of subjects; age at onset of the disease; treatment methods used; gender, reported treatment outcome were selected for final analysis. Results were categorised based on containment and noncontainment methods. Key words such as Perthes, avascular necrosis of the hip joint, treatment, orthosis, containment method, surgery, biomechanics, and gait analysis were selected for the final inclusion criteria.

### 2.1. Quality Assessment Tools and Data Extraction

First, the research design of the studies was determined. Then, the quality of their methodology was assessed by use of the Down and Black tool. Two expert reviewers were asked to evaluate the quality score of the methodology of each research study. The correlation between the reviewer's results was 0.87 for the Downs and Black test. It has been shown that the reliability and validity of this test is acceptable to be used in order to evaluate the quality of the methodology [15, 16].

### 2.2. Evaluating the Heterogeneity of the Results of Research Studies

Heterogeneity describes the difference between the results of various studies as a result of sampling error or due to present of a significant diversity of results. The heterogeneity of the results of the research studies was evaluated by use of *Q* test and by use of *I*². The *Q* test evaluates the heterogeneity that has a *λ*² distribution (with *n* degree of freedom, where *n* denotes the number of studies). *P* values less than 0.05 indicate the presence of significant heterogeneity. The *I*² was calculated based on the following equation:

(1) $$\begin{array}{c} I^{2}=\left[\frac{Q-df}{Q}\right]\times 100, \end{array}$$

where *Q* is *λ*² and df is the degree of freedom based on the number of studies. A rough guide used for heterogeneity in this study was as follows [15, 16]:

- 0–40: not important,
- 30–40: moderate heterogeneity,
- 50–70: substantial heterogeneity,
- 70–100: considerable heterogeneity.

### 2.3. Method of Assessment Used to Evaluate the Results of Various Treatment Approaches

A variety of different assessment methods have been used to evaluate the success of LCPD treatment. The most commonly used classification system in research studies is the classification system developed by the Paediatric Orthopaedic Society. This method classifies results as good, fair, or poor according to X-ray classification criteria of Stulberg et al. [9, 17, 18], Evans [1], and Kelly et al. [19] (Table 1). In Stulberg et al. method several radiographic parameters such as the spherically of femoral head, the length of femoral neck, the slop of acetabulum, and presence of coxa magna are evaluated. Based on this method, the hip joint is classified into one to five categorizes, which include spherical congruency (class I and II) and aspherical congruency (class III, IV, and V) [9].

The method employed by Mose (1980) determines the spherical measurement of the femoral head by means of transparent template with a circle drawn at 2 mm intervals. If the resulting bone outline is circular or deviates from a circular shape by less than 2 mm on both anteroposterior and lateral roentgenograms, the result is considered good. Deviation of 2 mm on either X-ray is considered fair, and a poor result determined to be a deviation of more than 2 mm [8].

The Wiberg centre edge angle is determined as the angle between the line connecting the lateral rim of acetabulum, the centre of femoral head, and a vertical line. This method is also commonly used to determine severity of LCPD.

Analysed studies were categorised by the following parameters: the number of subjects; the selected treatment methods; the final outcome of the treatment; follow-up duration; age at onset of the disease, gender.

## 3. Results

Initial application of the key search words generated a total of 100 articles from reviewed databases. Following application of inclusion criteria 50 papers were selected for final analysis. Analysis of papers determined four main themes which were dependant on the treatment method: containment; noncontainment; surgery, non-treatment. Based on the search strategy, 50 articles were found, most of which were prospective cohort study, case series, and case control (only one research study was randomized-control trial). The results of quality assessment of the research are summarised (Table 2), and the results of heterogeneity test summarised in Table 3.

One of the approaches used for treatment of LCPD is containment based method. In this treatment, various types of orthoses such as abduction brace, Scottish rite, and Birmingham orthoses have been used. The results of research studies describing this type of treatment showed that between 9 and 64.7% of subjects had a good outcome, based on Paediatric Orthopaedic Society classification system (Table 4).

Conventional callipers, Snyder slings, slings with crutches, and traction are other methods used as noncontainment approach. Although the number of subjects and follow-up duration of the selected research studies vary between the research studies, the final results were nearly the same (between 58.4 and 80% of the subjects had a good score of treatment).

Three different methods of surgical intervention have been described in LCPD. These include innominate osteotomy, femoral osteotomy, and a combination of both methods. Results indicate that between 44.4 and 92% of subjects with LCPD had a good score of treatment and did not show significant difference when compared to containment and noncontainment approaches.

Researched results of conservative treatment of LCPD, where no intervention is applied are shown in Table 7. Between 11 and 59% of the subjects in studies, shown in Table 7, had good final results. In contrast, form 16.7 to 75% of the subjects did not have a satisfactory outcome (poor results).

## 4. Discussion

Most studies examining treatment options in LCPD are of high quality and were of appropriate sample size (Table 2). In most studies, subjects were followed up by investigators for a long period of time ( from 4.5 to 22 years). Assessment methods used to evaluate the condition were the same in the most of the research studies. Heterogeneity tests also showed that the final results of different noncontainment treatment approaches did not illustrate important heterogeneity (Table 3). In contrast, the heterogeneity between the results of other methods of treatment was high which causes difficulty when establishing the effect of different treatment methods.

The main aims of treatment of LCPD are to prevent the further deformity of the femoral head, relief of symptoms, containment of the femoral head, and restoration of range of motion of the hip joint [11]. There are two main methods which have been used to reach to this goal: surgical and non-surgical approaches. Evidence to determine the best mode of treatment of the treatment of LCPD is inconclusive. Currently, most treatment approaches are based on preference, experience, and training of clinicians. Variability of factors which may affect disease process and the lack of knowledge regarding pathophysiology may also contribute to difficulties in establishing treatment guidelines.

A number of differing methods of treatment are also employed in the treatment of this disease including differing orthoses, the containment and noncontainment methods, and surgical and non-surgical intervention.

### 4.1. Orthotic Intervention

A variety of different orthoses are used in the treatment of LCPD (Tables 4 and 5). Orthoses may be categorized as ambulatory (e.g., the abduction orthosis, the Scottish rite orthosis, the Birmingham orthosis, traditional calipers, and the Snyder sling) or nonambulatory orthoses (such as traction and bed rest with sling).

Only one research paper was found that compared the results ambulatory versus nonambulatory treatment. Results of this paper indicated no outcome differences in the success of treatment [12] (Tables 4 and 5). The main aim of weight relief to protect the necrotic femoral head against body weight however, is not biomechanically valid as the muscle forces acting to support the joint in the specific location applied greater intra-articular pressure than forces produced during weight bearing [27]. Weight relieving methods may also produce additional adverse effects, such as muscular atrophy, osteoporosis, asymmetric reduction in thoracic kyphosis, urolithiasis, social-emotional problem, increasing health budget, and high cost of hospitalisation [24].

### 4.2. Comparison between the Containment and NonContainment Method

Due to the lack of consistency of methodology used by different research studies, it is not possible to conclude whether containment or noncontainment is the most effective method of treatment (Tables 4 and 5). Outcomes of treatment with containment and noncontainment methods were similar, although too much variation existed between age of onset, gender, and follow-up period. Kelly et al. (1980) have shown that the results not containing the femoral head (using either a harness or sling with crutches) were good or fair in 91% of the subjects [19, 28]. This would imply that the vast majority of patients with this disease can be successfully treated without any attempt to force the femoral head within the acetabulum. However, more evidence is required to allow data to be compared.

### 4.3. Surgical Intervention

Results comparing different methods of surgical intervention were inconclusive [23, 25, 26] (Table 7). However, following innominate osteotomy, the center edge and neck shaft angles, length of the limbs; range of abduction, and total range of motion have been demonstrated to normal. This procedure may offer improved angular positioning between the ball and the shaft of the femur due to reduced femoral growth plate involvement. This is also advantageous as scarring is minimised and less possibility of introducing limb shortening exists [20].

### 4.4. Comparison between Surgery and Using an Orthosis

Although treatment outcomes recorded are similar, the lack of consistency of methodologies used by different research studies makes it difficult to conclude whether surgical or non-surgical treatment is the most effective in management of this condition (Tables 4 and 7). The percentage of acetabular coverage, extent of lateral femoral subluxation, and age of the patients at onset of injury will influence the final results and must be taken into account during study design. One study compared orthotic treatment and surgical intervention in two groups of patients with LCPD by employing the same procedure [21]. The results of this research indicated similar outcomes following both methods of treatment. [21, 22, 29].

Two main aims for the treatment of LCPD are to decrease loads applied on the hip joint and increase the congruency of articular surface. The results of selected research studies focused in this paper show that there is little difference between the outputs of treatment methods. A further approach which has not been examined in detail would quantify the magnitude of the force applied on the hip joint over a period of time (force time integral of the vertical force applied on the hip joint during walking).

## 5. Conclusion

The aim of this paper was to examine the evidence of success of treatment methods used in Legg-Calve-Perthes disease (LCPD) to assist in determining appropriate pathways for treatment.

Evidence relating to the effectiveness of the treatment method used was found to be conflicting. Most treatments were based on preference, experience, and training of surgeons and clinical staff. Research using different methods of treatment were difficult to compare as different methods of screening and follow-up periods were employed in each study which used subjects of varying ages.

Greater research of appropriate design is required to facilitate comparison of outcome measurements recorded as a result of different treatment options.

## Figures and Tables

**Table 1:** Reported assessment methods used to categorise severity of LCPD disease.

Score	Mose method [8]	Wiberg centre edge angle (Stulberg et al.) (1981) [9] degree
Good	0	20
Fair	2	15–19
Poor	>2	<15

**Table 2:** The results of quality assessment of methodology of various research studies (total number of studies was 50).

Treatment approaches	Reporting (total score 10)	External validity (total score 3)	Internal validity, bias (total score 7)	Internal validity, confounding (total score 7)
Containment methods	9 (7–10)	3	4 (3–6)	5 (4–6)
Noncontainment methods	8 (7–10)	3	4 (3–6)	6 (5–7)
Nontreatment	9 (8–10)	3	4 (3–6)	5 (4–7)
Surgery methods	9 (8–10)	3	4 (3–6)	5 (4–7)

**Table 3:** Heterogeneity of research studies.

Treatment approaches	*P* value of *λ*²	*I* ^2^ index value	Degree of heterogeneity
Containment methods	0–0.081	51.8–95.8	Substantial to considerable
Noncontainment methods	0.012–0.164	0.28–72.7	Not important to substantial
Nontreatment	0–0.183	38.14–96.66	Not important to considerable
Surgical methods	0	83–87.82	Considerable

**Table 4:** Results of containment research studies.

Research	Number of subjects	Gender	Age (year)	Follow-up period (year)	Method	Results (%)
Martinez et al. [20]	60	49 male, 11 female	5–11	1.5	Abduction orthosis	60.3 good, 30.9 fair, 8.8 poor
Herndon et al. [21]	17	No information	7	No information	Brace	64.7 good, 17.6 fair, 17.6 poor
Meehan et al. [22]	34	No information	8	6.7	Scottish Rite orthosis	9 good, 26 fair, 65 poor
Stulberg et al. [19]	88	76 male,12 female	7.5	47.3	Bed rest with sling	26 good, 25 fair, 49 poor
Harrison et al. [13]	233	160 male, 53 female	No information	5.8	Birmingham splint	57 good, 38 fair, 5 poor

**Table 5:** Results of noncontainment studies.

Research	Number of subjects	Gender	Age (year)	Follow-up period (year)	Method	Results (%)
Evans et al. [1]	24	male	3–8	6.75	Calliper	62.5 good, 20.8 fair, 16.7 poor
Evans et al. [1]	24	male	3–8	4.75	Snyder sling	58.4 good, 16.6 fair, 25 poor
Kelly et al. [23]	80	Both	8–10	22.4	Sling with crutches	80 good, 11.25 fair, 8.75 poor
Herndon et al. [21]	37	No information	3.5–11	7.4	Traction followed by Ischial weight bearing orthosis	32.4 normal, 47.6 good, 10 fair, 10 poor

**Table 6:** The results of various research studies based on surgical operations.

Research	Number of subjects	Gender	Age (year)	Follow-up period (year)	Method	Results (%)
Robinson et al. [14]	27	Not reported	6.33	5–16	Innominate osteotomy	92 good, 0 fair, 8 poor
Bellyei and Mike [15]	30	Not reported	Not reported	Not reported	Femoral osteotomy	57 good, 23 fair, 20 poor
Bellyei and Mike [15]	19	Not reported	6.9	Not reported	Femoral osteotomy	63.15 good, 10.5 fair, 26.3 poor
Paterson et al. [24]	27	Not reported	5.9	9.3	Innominate osteotomy	56 good, 41 fair, 4 poor
Lloyd et al. [25]	16	Not reported	6	4–9	Femoral osteotomy	62.5 good, 25 fair, 12.5 poor
Lloyd et al. [25]	18	Not reported	6	Not reported	Femoral osteotomy	44.4 good, 22.2 fair, 33.4 poor

**Table 7:** The results of conservative treatment.

Research	Number of subjects	Gender	Age (year)	Follow-up period (year)	Results (%)
Catteral [26]	46	37 male, 9 female	4.5	4	59 good, 20.8 fair, 16.7 poor
Lloyd et al. [25]	75	Not reported	Not reported	Not reported	58.4 good, 16.6 fair, 25 poor
Catteral [7]	36	Not reported	6.65	Not reported	11 good, 31 fair, 58 poor
Catteral [26]	8	6 male, 2 female	4.5	4	25 fair, 75 poor
